# Interfacial Water and Microheterogeneity in Aqueous Solutions of Ionic Liquids

**DOI:** 10.1021/acs.jpcb.1c10961

**Published:** 2022-06-01

**Authors:** Cettina Bottari, László Almásy, Barbara Rossi, Brenda Bracco, Marco Paolantoni, Andrea Mele

**Affiliations:** † Elettra Sincrotrone Trieste, S.S. 114 km 163.5, Basovizza, 34149 Trieste, Italy; ‡ Institute for Energy Security and Environmental Safety, Centre for Energy Research, Konkoly-Thege Miklós út 29−33, 1121 Budapest, Hungary; § Department of Chemistry, Biology and Biotechnology, University of Perugia, Via Elce di Sotto 8, 06123 Perugia, Italy; ∥ Department of Chemistry, Materials and Chemical Engineering “G. Natta”, Politecnico di Milano, 20133 Milano, Italy

## Abstract

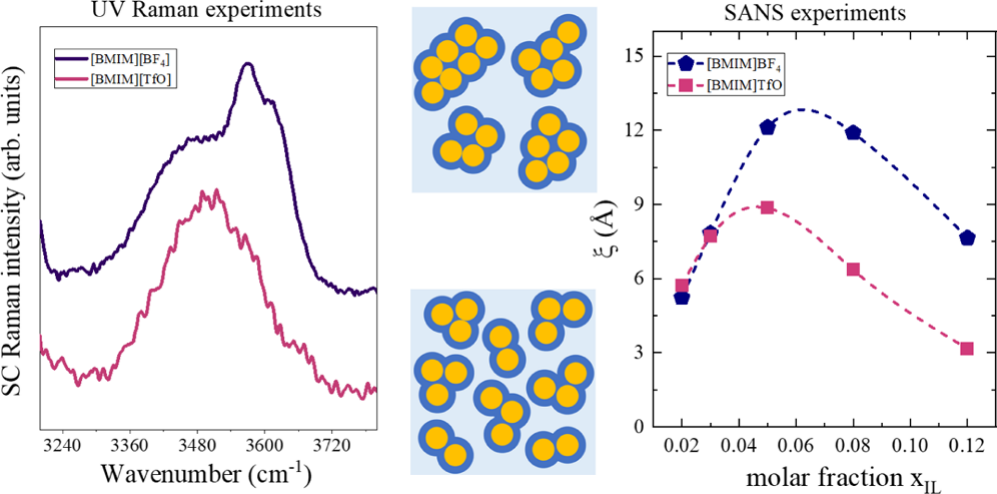

In this work, aqueous solutions of two prototypical ionic liquids (ILs), [BMIM][BF_4_] and [BMIM][TfO], were investigated by UV Raman spectroscopy and small-angle neutron scattering (SANS) in the water-rich domain, where strong heterogeneities at mesoscopic length scales (microheterogeneity) were expected. Analyzing Raman data by a differential method, the solute-correlated (SC) spectrum was extracted from the OH stretching profiles, emphasizing specific hydration features of the anions. SC-UV Raman spectra pointed out the molecular structuring of the interfacial water in these microheterogeneous IL/water mixtures, in which IL aggregates coexist with bulk water domains. The organization of the interfacial water differs for the [BMIM][BF_4_] and [BMIM][TfO] solutions, being affected by specific anion–water interactions. In particular, in the case of [BMIM][BF_4_], which forms weaker H-bonds with water, the aggregation properties clearly depend on concentration, as reflected by local changes in the interfacial water. On the other hand, stronger water–anion hydrogen bonds and more persistent hydration layers were observed for [BMIM][TfO], which likely prevent changes in IL aggregates. The modeling of SANS profiles, extended to [BPy][BF_4_] and [BPy][TfO], evidences the occurrence of significant concentration fluctuations for all of the systems: this appears as a rather general phenomenon that can be ascribed to the presence of IL aggregation, mainly induced by (cation-driven) hydrophobic interactions. Nevertheless, larger concentration fluctuations were observed for [BMIM][BF_4_], suggesting that anion–water interactions are relevant in modulating the microheterogeneity of the mixture.

## Introduction

The well-known capability of imidazolium-based, aprotic ionic liquids (AILs) to generate polar and nonpolar domains is the major source of the structural heterogeneities commonly seen as a fingerprint of these systems.^1−5^ Such a characteristic behavior, also shared by piperidinium-based AILs,^6^ stems from the interplay between long-range Coulombic interactions and short-range H-bonding, van der Waals, and solvophobic ones.^7^ Commonly, the extent of heterogeneous organization increases with increasing the length of the cation alkyl chains due to the enhancement of hydrophobic effects that favors the separation of polar and apolar regions.^7^

Water addition can drastically change the inhomogeneous organization of the ILs and, in turn, their macroscopic properties, thus representing a suitable way to tune their behavior in view of specific applications.^8−10^ At low hydration levels, the nanodomain structuring on ILs is largely maintained, the water molecules being dispensed into the polar regions of the IL network, interacting preferentially with the ionic moieties.^7,9^ Increasing water concentration favors water clustering, with the possible formation of specific architectures such as interconnected water channels, confined water domains, or reverse micelles, depending on the IL nature and concentration.^9,11^ At higher water contents, generally corresponding to water mole fractions *x*_w_ > 0.9, water molecules produce extended percolating networks, within which IL species self-organize based on their polar/apolar character.^7^ The formation of a bicontinuous structure of interpenetrating percolating networks of both water and IL was also considered.^12^ In this water-rich domain, IL/water mixtures can exhibit enhanced spatial (and dynamical) heterogeneities, also due to the possible formation of specific IL aggregates (i.e., micellar-like) dispersed in the aqueous medium.^7,9^ Commonly, the dissociation of ILs in small ion clusters requires larger water contents, starting from *x*_w_ ∼ 0.95,^12^ yet ion pairs are expected to exist even in more diluted conditions.^10^

Crucial information on the heterogeneities of IL/water mixtures was obtained by small-angle neutron scattering (SANS) experiments on prototypical aqueous solutions of [BMIM][BF_4_].^13^ Bowers et al., from SANS, surface tension, and conductivity measurements, suggested that starting from diluted conditions, polydisperse spherical [BMIM][BF_4_] aggregates form above a critical aggregation concentration (CAC) located at around IL mole fraction *x*_IL_ ∼ 0.015 (*x*_w_ ∼ 0.985).^14^ Katayanagi et al. analyzing thermodynamic data identified a transition in the “solution structure” just at *x*_IL_ = 0.015, which was related to the beginning of either direct or water-mediated ion association.^15^ A subsequent work by Almásy et al. demonstrated that the SANS profiles of [BMIM][BF_4_]/water mixtures can be well interpreted in terms of strong concentration fluctuations within the range of 0.02 < *x*_IL_ < 0.16 and that the greatest heterogeneous structuring occurs at *x*_IL_ ∼ 0.075 (about 50% in volume) when the system approaches the phase separation. It was also remarked how the formation of micellar-like aggregates could be neither excluded nor confirmed due to the dominant scattering contribution arising from concentration fluctuations.^16^ Analogous heterogeneous mixing was observed in other binary organic solvent/water and IL/organic solvent mixtures.^17−19^ On the other hand, according to the SANS study of Kusano et al.,^20^ [BMIM][Cl] and [BMIM][Br] mix homogeneously with water due to the strong hydration capability of the anions. Remarkably, in the case of [BMIM][NO_3_]/water mixture, SANS experiments indicated the presence of confined water within the IL network—referred to as “water pockets” (WP)—starting from rather diluted conditions *x*_IL_ ∼ 0.05 to about *x*_IL_ ∼ 0.30,^21^ further evidencing that, even for relatively small cations, a variety of organization features might exist in the water-rich domain. Concerning the aggregation of pyridinium-based ILs in water, clear signatures of interparticle interactions were not observed by SANS experiments for 1-butylpyridinium and 1-hexylpyridinium chlorides, while a distinct structural peak was observed for longer-chain cations.^22^

The full concentration range of [BMIM][BF_4_]/water mixtures was explored by Gao and Wagner^13^ based on SANS and literature results. It was argued that water molecules remain dispersed within the IL network up to *x*_w_ ∼ 0.7 (*x*_IL_ ∼ 0.3), while for higher *x*_w_ values, a microphase separation would take place due to the formation of water nanoclusters among the percolating IL network. These water pockets progressively grow with further water addition, and a phase inversion will occur for *x*_w_ > 0.84 (*x*_IL_ <0.16) due to the formation of micelle-like IL aggregates dispersed in the percolating network of water. Additionally, the full dissociation of IL species would occur only for water mole fractions larger than *x*_w_ ∼ 0.985 (*x*_IL_ ∼ 0.015), as suggested by Katayanagi et al.^15^ Mixtures in which salt/water ratios are larger than one by volume (*x*_IL_ > 0.075 for [BMIM][BF_4_]) were recently conceptualized as a promising class of systems in different application areas and specifically denoted as “water-in-salt” mixtures.^9^ It has been suggested that the water structuring in BMIM-based IL mixtures might be tuned by changing the nature of the anion, which also determines the IL hydrophilic/hydrophobic character and its full miscibility with water.^23,24^ In particular, while [BMIM][BF_4_] and [BMIM][TfO] are hydrophilic and fully miscible with water, the replacement of BF_4_^–^ with TfO^–^ would make the system more hydrophobic, promoting water percolation. Overall, the specific aggregation properties of AIL/water mixtures formed by short-chain cations strongly depend on anion type and water contents and are still under debate.

The mesoscopic picture on IL/water mixtures derived by SANS experiments can be flanked by an atomistic point of view on their intermolecular interactions achieved by vibrational spectroscopies,^25^ as they are sensitive to modulations of local potentials around a probing oscillator. Regarding the prototypical [BMIM][BF_4_]/water system, Fazio et al. showed that a fraction of bulk-like water in the mixture grows up from *x*_w_ ∼ 0.5 and it starts to affect the anion–cation interaction at *x*_w_ ∼ 0.7.^26^ The vibrational study of Jeon et al. suggested the occurrence of structural changes in the water-rich domain, at around *x*_w_ ∼ 0.93 and 0.98, as inferred from the concentration dependence of the signals of the anion, cation, and water as well.^27^ It was proposed that cation aggregation (or micellization) occurs at *x*_IL_ > 0.02 (*x*_w_ < 0.98), leading to modifications of the water structuring. Indeed, MD simulations indicate that IL aggregation takes place at *x*_w_ > 0.8, attaining its maximum extent at *x*_w_ ∼ 0.9–0.95.^28^ On the other hand, analyzing the OD stretching band in the IR spectrum of [BMIM][BF_4_]/D_2_O mixtures, Zheng et al.^29^ suggested that IL aggregates and ion pairs dissociate when *x*_w_ > 0.9. As a representative case of pyridinium-based IL, aqueous mixtures of 1-butylpyridinium tetrafluoroborate ([BPy][BF_4_]) were studied by the combined use of IR and DFT methods by Wang et al.^30^ These authors inferred that the substitution of BMIM with BPy would impact the overall H-bonding of the mixtures, basically due to the different acidity and steric hindrance of the aromatic C–H groups in the two cations.

We remark that the characteristic spectral features of the OH stretching of water molecules dispersed within concentrated ILs can be observed by vibrational spectroscopies.^31−33^ In these conditions, the spectral features were commonly related to water–anion interactions, albeit some discrepancies persist in literature about the specific geometry of the clusters.^29,31,33^ Nevertheless, drawing a clear picture of the water state in the water-rich concentration range (*x*_w_ > 0.8) is much more challenging, as hydrating water molecules coexist with bulk-like ones and the OH stretching band broadens significantly, encompassing water–water and water–IL contributions. Considering the microheterogeneous character of the system, this hydration water can be conjectured as a sort of “microinterface”, separating bulk water and IL-rich domains, with features related to IL clustering and/or concentration fluctuations, which would depend on the IL content. Thus, gaining insights into this interfacial water appears crucial for a molecular-level understanding of the physicochemical and solvation properties of the mixtures but difficult to achieve.

Here, AILs/water systems were investigated in the water-rich regime when IL and water mesoscopic domains are expected to form. Aqueous solutions of [BMIM][BF_4_] and [BMIM][TfO] were analyzed by SANS and UV Raman spectroscopy to relate their microheterogeneous character with the molecular state of water at the “microinterface”. The SANS investigation was further extended to [BPy][BF_4_] and [BPy][TfO]/water mixtures to highlight the effects induced by the cation on the microheterogeneous mixing. The atomistic view was derived by UV Raman experiments based on the approach proposed by Ben-Amotz et al.^34,35^ The method, when coupled with multivariate curve resolution (MCR) analysis, was proven suitable to isolate from the OH stretching Raman band of water, the so-called solute-correlated spectrum (SC), which contains the spectral contribution of those water molecules affected by the solute (hydrating water).^34−38^ Here, UV Raman SC spectra were obtained by a direct spectral subtraction procedure,^35,36,38^ providing novel molecular insights into the interfacial water in microheterogeneous IL/water mixtures.

## Methods

### Preparation of Ionic Liquids/Water Solutions

**Scheme 1:**
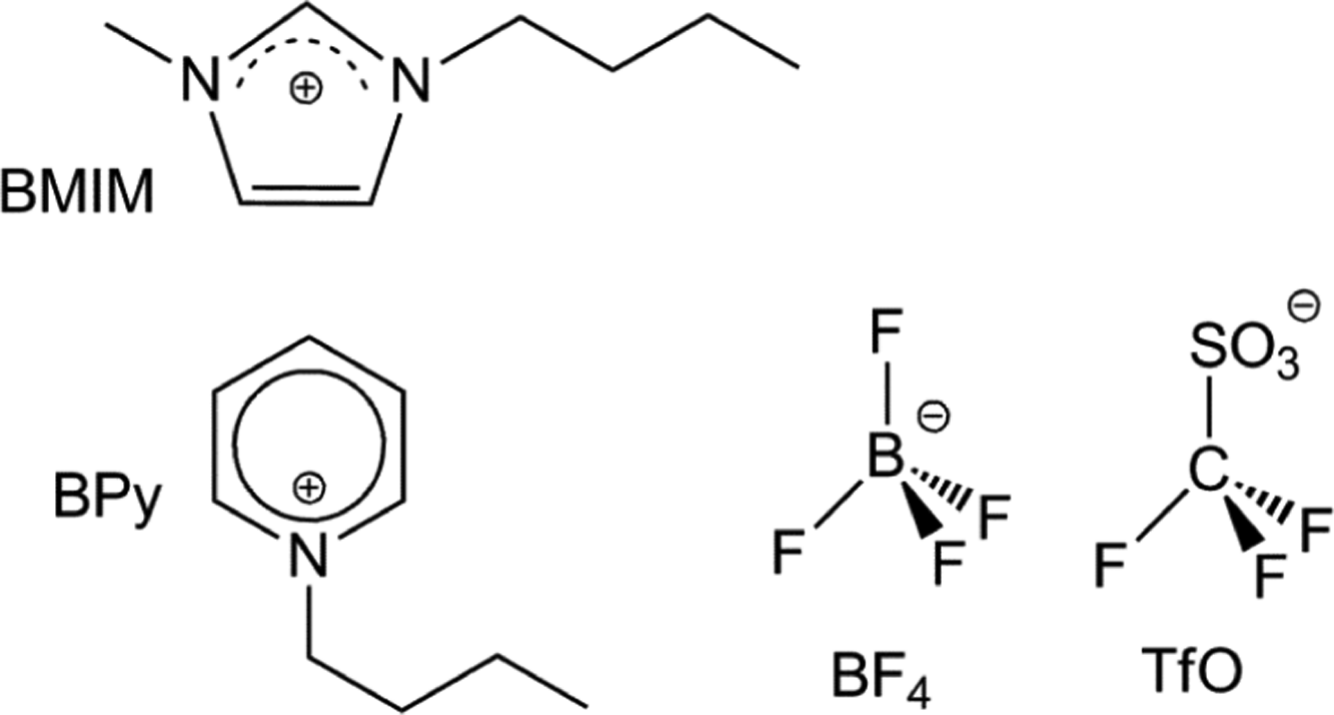
Molecular Formulae and Labels of the Cationic and Ionic Components of the Examined ILs

1-Butyl-3-methylimidazolium tetrafluoroborate ([BMIM][BF_4_]), 1-butyl-3-methylimidazolium triflate ([BMIM][TfO]), 1-butylpyridinium tetrafluoroborate ([BPy][BF_4_]), and 1-butylpyridinium triflate([BPy][TfO]) were purchased from IoLiTec with a purity of 99%. The molecular structures of the components of ILs are displayed in Scheme 1. For UV Raman experiments, ILs/water solutions were prepared using high-purity water deionized through a MilliQ water system (>18 MΩ cm resistivity). Heavy water of 99.3 atom % deuterium content was used for preparing the solutions for SANS experiments to increase the contrast in the scattering length densities between IL and water, thus improving the experimental accuracy. The aqueous solutions of ILs were prepared at different IL mole fractions, , where *n*_IL_ and *n*_water_ are the number of moles of IL and water, respectively.

### SANS Measurements

SANS experiments were carried out using the Yellow Submarine diffractometer operating at the Budapest Neutron Center.^39^ Samples were placed in 2 mm thick Hellma quartz cells. The temperature was controlled within 0.1 K using a Julabo FP50 water circulation thermostate. The range of scattering vectors *q* was set to 0.038–0.38 Å^–1^. The *q* value is defined as *q* = 4π/λ sin θ, where 2θ is the scattering angle. To have access to the whole range of *q*, we used two different configurations with sample-detector distances of 1.15 and 5.125 m and the incident neutron wavelength was set to 4.4 Å.

The raw data have been corrected for sample transmission, scattering from an empty cell, and room background. Correction to the detector efficiency and conversion of the measured scattering to absolute scale was performed by normalizing the spectra to the scattering from a light water sample.

### UV Raman Measurements

UV Raman experiments were carried out by exploiting the synchrotron-based UV Raman setup available at the BL10.2-IUVS beamline of Elettra Sincrotrone Trieste (Italy).^40^ The employed experimental apparatus was proven suitable for the acquisition of high-quality UV Raman spectra of aqueous solutions in the CH/OH stretching region (2600–3900 cm^–1^).^43^ The Raman spectra of ILs/water solutions were recorded using excitation wavelengths in the deep UV range at 248 and 266 nm. These excitation conditions were chosen to obtain suitable Raman signals at all of the concentrations considered, minimizing the fluorescence background and with the best features in terms of the spectral resolution and signal-to-noise ratio. In particular, the 248 nm excitation wavelength was employed to avoid the interference of a fluorescence background observed with the 266 nm excitation for the [BPy][TfO] samples, likely due to minor impurities. The 248 nm excitation wavelength, provided by the synchrotron source (SR), was set by regulating the undulator gap and using a Czerny–Turner monochromator (Acton SP2750, Princeton Instruments, Acton, MA) equipped with a holographic grating with 1800 grooves per mm for monochromatizing the incoming SR. The excitation radiation at 266 nm was provided by a CryLas FQSS 266-Q2 diode-pumped passively Q196 switched solid-state laser. The UV Raman spectra were collected in back-scattered geometry using a single pass of a Czerny–Turner spectrometer (Trivista 557, Princeton Instruments, 750 mm focal length) and detected with a CCD camera. The spectral resolution was set at 1.6 and 2 cm^–1^/pixel for the measurements with 266 and 248 nm as excitation wavelengths, respectively. The calibration of the spectrometer was standardized using cyclohexane (spectroscopic grade, Sigma-Aldrich). The power of the beam on the sample was measured to be a few microwatts; any possible photodamage effect due to prolonged exposure of the sample to UV radiation was avoided by continuously spinning the sample cell during the measurements. Solute-correlated (SC) spectra^34,35^ were extracted from the OH stretching distribution by a direct spectral subtraction procedure as the difference between the spectrum of the mixture and a properly rescaled spectrum of neat water. The rescaling factor is determined in such a way that the resulting difference distribution is non-negative and with the minimum area.^38^

## Results and Discussion

### Microscopic Viewpoint: UV Raman Experiments

Water-rich mixtures of [BMIM][BF_4_] and [BMIM][TfO] were examined as a function of concentrations (0.02 < *x*_IL_ < 0.16) by UV Raman spectroscopy, focusing on the OH stretching spectral region (3000–3800 cm^–1^), which is particularly sensitive to the H-bonding configurations of water.^41−43^ Several IR and Raman studies have already been performed to characterize these mixtures;^26,27,29,31,33,44,45^ however, a clear description of the hydration properties of the two systems in the presence of a large amount of water (*x*_w_ > 0.8) is still missing.

**Figure 1:**
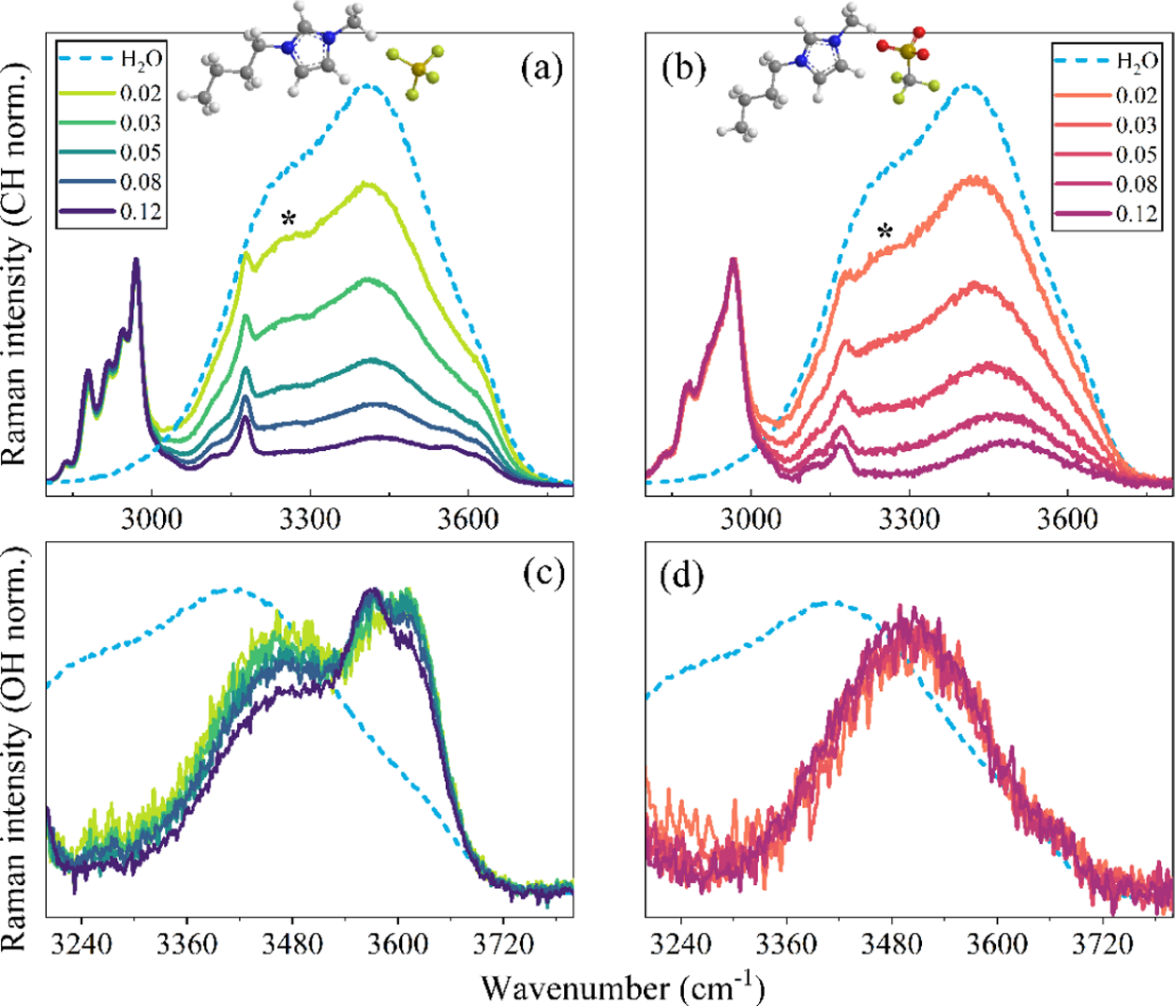
UV Raman spectra of [BMIM][BF_4_] (a) and [BMIM][TfO] (b) aqueous solutions at different IL mole ratios normalized on CH stretching signals (2800–3000 cm^–1^); the spectrum of neat water is reported in the same graphs (dot line). The symbol * marks the spectral signature at around 3200 cm^–1^ due to the ice-like component of bulk water. Solute-correlated (SC) UV Raman spectra obtained for [BMIM][BF_4_] (c) and [BMIM][TfO] (d) after rescaling on the maximum intensity; the Raman spectrum of neat water is shown in the same panels (dot line).

The Raman spectra (2800–3800 cm^–1^) for the [BMIM][BF_4_] and the [BMIM][TfO] aqueous solutions are reported in Figure 1a,b, respectively, together with the spectrum of pure water. The spectra of the mixtures were normalized on the CH stretching signals (2800–3000 cm^–1^), while the spectrum of pure water was arbitrarily rescaled. For both systems (Figure 1a,b), the broad OH stretching band (3000–3800 cm^–1^) shows a decrease in intensity and a global shift toward higher wavenumbers with the increase of IL content. This blue shift indicates the general weakening of the H-bond interactions and destructuring of the H-bond network typical of water,^42^ owing to the substitution of water–water H-bonds with specific water–anion ones. Modifications of the OH stretching band are mainly ascribed to changes of the H-bonding state of water acting as a H-donor due to the establishment of new O–H···X interactions with the anions.^44^ Water–anion H-bonds are expected to be relevant even at high IL contents when water–cation correlations become less significant.^28,46^

Specific information about the interfacial water was extracted from the solute-correlated spectra (SC) by a direct spectral subtraction procedure, considering the spectrum of pure water as a reference for the bulk water in the mixture.^34−38^ The resulting SC-UV Raman spectra are compared in Figure 1c,d for the [BMIM][BF_4_] and [BMIM][TfO] solutions, respectively, and after normalization to the maximum intensity. The SC-UV Raman spectra emphasize the (minimum) perturbation of the water structure as induced by the solute.^34−38^ We remark that in the reported spectral region (3240–3720 cm^–1^) no direct spectral contributions arising from the cation and anion are present. For both systems, the SC spectra are located at higher wavenumbers compared to the spectrum of pure water, confirming that, under the influence of the anions, hydrating water molecules form weaker H-bonds than in the bulk.

**Figure 2:**
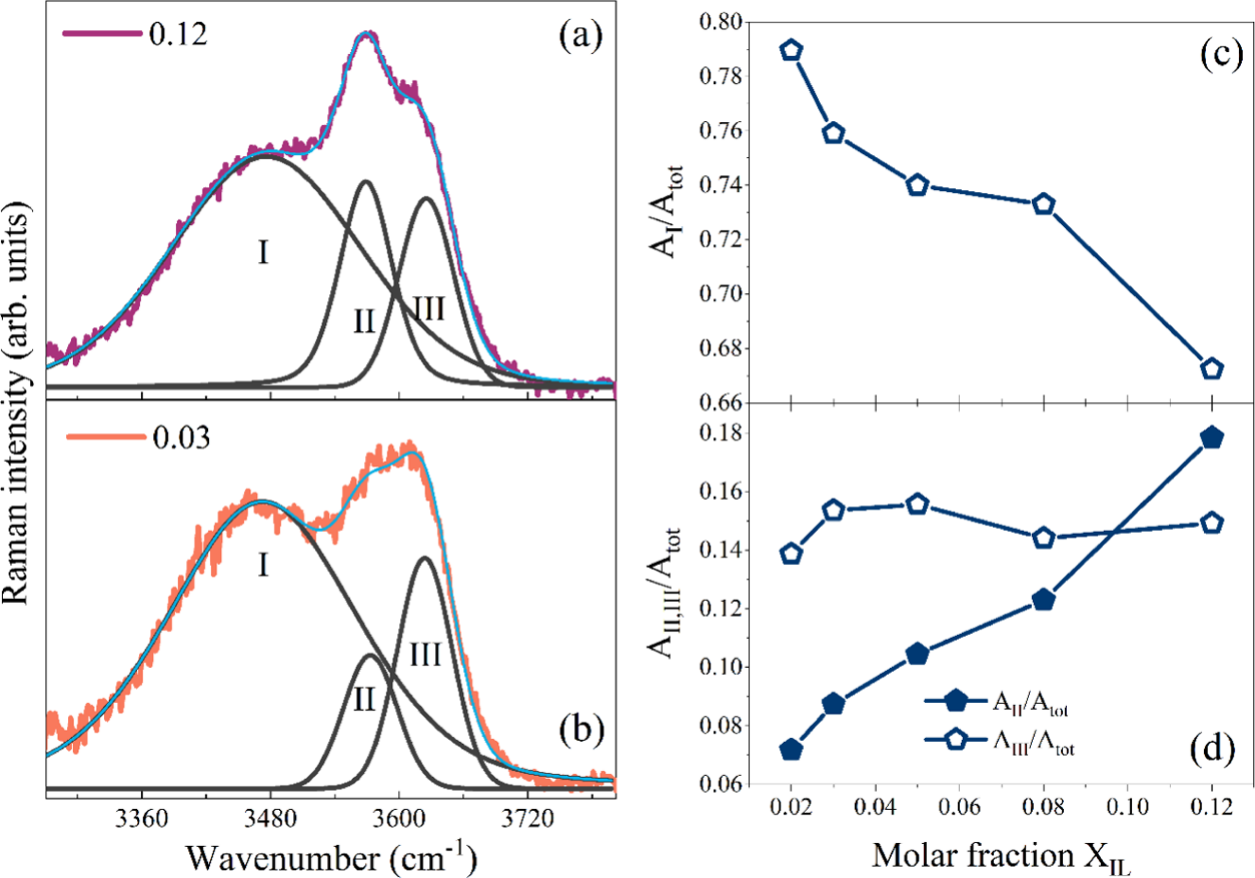
Results of a fitting procedure using three Voigt functions to reproduce the SC-UV Raman spectra of [BMIM][BF_4_] solutions at two representative concentrations: *x*_IL_ = 0.12 (a) and *x*_IL_ = 0.03 (b). The relative peak areas *A*_I_/*A*_tot_, *A*_II_/*A*_tot_, and *A*_III_/*A*_tot_ corresponding to components I, II, and III, respectively (normalized to the total area *A*_tot_), are also reported as a function of *x*_IL_ (c, d).

**Figure 3:**
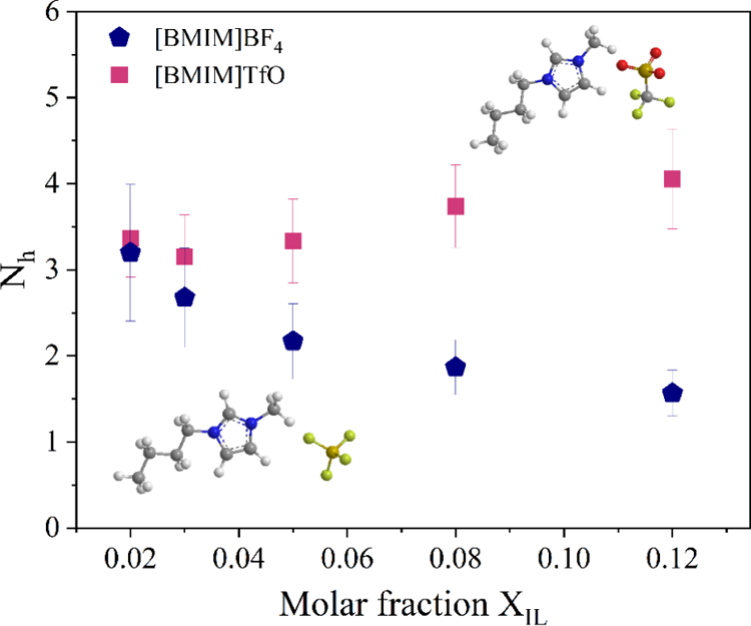
Apparent hydration number *N*_h_ (number of water molecules perturbed by the solute) derived for aqueous solutions of [BMIM][BF_4_] (blue pentagons) and [BMIM][TfO] (violet squares).

The SC spectra of [BMIM][BF_4_] solutions (Figure 1c) clearly show the presence of different subcomponents whose intensity strongly depends on concentration. Thus, a partial redistribution of different types of water–anion contacts is taking place in the explored concentration domain. The resulting SC spectra can be reproduced by means of a curve-fitting procedure using three components located at ∼3475, 3565, and 3625 cm^–1^, here referred to as components I, II and III, respectively. The results of the spectral decomposition are reported in Figure 2a,b for two representative samples (*x*_IL_ = 0.03 and 0.12). The two high-frequency components II and III relate to water molecules involved in the formation of rather weak H-bonds with BF_4_ anions.^31,44^ The Raman spectra of water dispersed into concentrated [BMIM][BF_4_] samples show two bands at 3565 and 3643 cm^–1^ due to the symmetric ν_1_ and asymmetric ν_3_ stretching vibrations, respectively.^47^ These have been assigned to water molecules interacting with two different anions (A^–^), leading to the formation of a “symmetric” adduct (A^–^···H–O–H···A^–^) with nearly 2 equivalent H-bonds.^47^ In the Raman spectrum, the symmetric ν_1_ band is significantly more intense than the asymmetric one ν_3_, with an intensity ratio of *I*_ν_1__/*I*_ν_3__ ∼ 5.6; an opposite situation occurs for the IR spectrum: *I*_ν_1__/*I*_ν_3__ ∼ 0.5.^31,47^ Thus, based on their positions and relative intensities in our SC-UV Raman spectra, component II (∼3565 cm^–1^) can be attributed to the ν_1_ mode of the mentioned “symmetric” water configuration, characterized by a H-bonding energy of about 9.5 kJ/mol.^31^ These results demonstrate that the water structures (A^–^···H–O–H···A^–^), previously detected at low water concentrations,^31^ continue to exist also in the water-rich domain (Figure 2c) when IL aggregates are dispersed in the aqueous medium.^13,28^ Component III (∼3625 cm^–1^) can be mainly attributed to water molecules interacting with only one anion.^48^ In that case, both OH groups might be doubly H-bonded with the same anion or, more likely, the formation of asymmetric configurations should be considered (O···H–O–H···A^–^). In these configurations, one OH group interacts with one anion, while the other OH is H-bonded to a second water molecule. Asymmetric ν_3_ stretching vibrations of A^–^···H–O–H···A^–^ adducts, expected at ∼3640 cm^–1^, might also contribute to component III.^47^ Finally, component I (∼3475 cm^–1^) can be assigned to water–water H-bonds involving water molecules already associated with the anion.^26,44^ Considering their spectral location, these water–water H-bonds are still significantly weaker than those formed, in average, in neat water, whose Raman spectrum presents two main components at ∼3200 and ∼3400 cm^–1^ assigned to tetrahedral (ice-like) structures and more distorted configurations, respectively.^42^ Nevertheless, in the Raman spectra of Figure 1a, the persistence of a spectral signature at around 3200 cm^–1^ (labeled with *), due to the ice-like component of bulk water, can be inferred even for the more concentrated [BMIM][BF_4_] solutions. Based on the ratio between the area of the SC spectrum (*A*_SC_) and that of the solution spectrum (*A*_SOL_), a rough evaluation of the fraction of hydration water is attempted. As a result, about 80% of bulk water is estimated to be still present in the *x*_IL_ = 0.12 solution. Thus, the three components observed in the SC spectra (Figure 1c) specifically account for the fraction of water molecules located in the “microinterface” between IL clusters and the percolating bulk-like water network.^28^ From the fraction of hydration water, an approximate estimate of the (minimum) number of perturbed water molecules per solute—here referred to as the apparent hydration number (*N*_h_)—can also be obtained.^36,49^ This is given by *N*_h_ = *A*_SC_*A*_SOL_^–1^*f*^–1^, where *f* is the solute-to-water mole ratio, *f=x*_IL_(1 – *x*_IL_)^−1^. As evidenced in Figure 3, in diluted conditions (at least), about three water molecules per anion are structurally affected. The average number of water molecules per solute (*f*^–1^) goes from 50 to 7 in the explored concentration range. The average *N*_h_ for [BMIM][BF_4_] (blue pentagons) decreases with *x*_IL_, indicating the occurrence of anion clustering. This is in line with the trends depicted in Figure 2 that clearly indicate structural modifications of the interfacial water. In particular, Figure 2c,d shows that, with the increase of *x*_IL_, the relative area of component I reduces and that of component II increases, while the contribution of component III remains almost constant. This evidences that the interfacial water involves an increasing fraction of A^–^···H–O–H···A^–^ structures at higher IL concentrations, consistently with anion self-association.

Thus, Raman findings reveal an overall decrease of the anion exposition to water upon increasing of *x*_IL_, pointing out the presence of IL aggregates and their modulation (i.e., size and/or distribution) within 0.02 < *x*_IL_ < 0.12. Since the [BMIM][BF_4_] is expected to fully dissociate only at *x*_IL_ < 0.02,^15^ the observed modifications would take place among ion aggregates (at least ion pairs). The occurrence of changes in aggregation properties of the [BMIM][BF_4_]/water mixtures agrees with previous MD simulations.^28,50^ Here, we demonstrate that these changes can be detected as modulations of the interactions experienced by the interfacial water. Evaluating the CH stretching trends of the groups belonging to the imidazole ring (signals at 3050–3200 cm^–1^), we do not observe any relevant shift within 0.02 < *x*_IL_ < 0.12, in line with previous IR investigations,^27^ suggesting that the strong anion–cation interactions (ion pairs) remain rather unperturbed. Moreover, the relatively strong concentration dependence, reported for the CH_3_ and CH_2_ stretching bands at 2800–3000 cm^–1^ of the butyl moiety,^27^ is not confirmed by our data, which instead suggest that also the local environment around the hydrophobic portions of the cations is not strongly affected by the aggregation process. These results are consistent with the presence of ion aggregates, stabilized by cation–cation hydrophobic contacts and anion–water H-bonds, forming nanodomains dispersed in the water medium, possibly with a micellar-like character.^13^ These aggregates and their hydration properties should play a role in determining the concentration fluctuation features evidenced by SANS experiments.

A significantly different scenario emerged from the analysis of the SC-UV Raman spectra of [BMIM][TfO] (Figure 1d). SC spectra show one single component positioned at about 3500 cm^–1^. The position and shape of the band do not depend on *x*_IL_, indicating that the structure of the interfacial water does not change with concentration. Basically, each spectrum is given by two types of water environments at all concentrations: bulk-like and hydration water, represented by bulk and SC spectra, respectively. The global blue shift observed for the solution spectra (Figure 1b) can then be rationalized considering that the relative contribution of interfacial water (SC spectrum) increases with IL concentration. More specifically, the band in the SC spectrum can be assigned to the symmetric stretching ν_1_ of water molecules interacting with two different TfO anions (A^–^···H–O–H···A^–^).^31^ Anyhow, the assignment to water molecules H-bonded to a single TfO anion cannot be excluded. The position of the band indicates that the strength of water–anion H-bonds increases significantly on going from BF_4_ to TfO. Based on a frequency-energy empirical correlation, the water–TfO H-bonding energy of about 16 kJ/mol can be estimated.^31^ As reported in Figure 3, an apparent hydration number *N*_h_ of about 3.5 is found, suggesting that, in diluted conditions, the number of water–anion H-bonds formed by TfO and BF_4_ are similar. Nevertheless, for the [BMIM][TfO] mixture, neither the number of perturbed water molecules nor the corresponding SC spectral distribution depends on concentration within experimental errors. Thus, in this case, IL clustering processes were not evidenced, and the anion solvation is rather unperturbed within the 0.02 < *x*_IL_ < 0.12 range. This agrees with the reported propensity of TfO to form strong H-bonds with both water and cation.^51^ A recent study on [Emim][TfO]/water mixtures further emphasized that TfO tends to interact strongly with water while maintaining a persistent anion–cation interaction, even in high diluted conditions, for the particular stability of the hydrated ion-pair dimers.^52^ Considering the CH stretching signals, also in this case, no meaningful modifications were detected in the 0.02 < *x*_IL_ < 0.12 range, in line with previous studies on the related [EMIM][TfO] compound.^52^

Thus, together with literature results, our Raman analysis in the water-rich domain of [BMIM][TfO] mixtures points out the existence of persistent IL–water adducts, as reflected by cation CH stretching bands and anion hydration features. As a result, the amount of interfacial water in this system grows up significantly with *x*_IL_. The total number of water molecules per solute is 7.3 at *x*_IL_ = 0.12; thus, the estimated *N*_h_ ∼ 4 (Figure 3) indicates that only ∼45% of bulk water is present at this mole fraction. Likely, the stronger water–anion H-bonds contribute to reducing the aggregate growth more efficiently in the [BMIM][TfO] mixtures compared to that in [BMIM][BF_4_]. Thus, the two systems clearly exhibit a different tendency to aggregate, related to the nature of the anion. The replacement of BF_4_ with TfO modifies the properties of the interfacial water and its amount.

In summary, three important aspects emerge from the analysis of the UV Raman data: (i) the IL–water interactions are modulated by the specific structural features and H-bond properties of the anions, with the BF_4_ acting as a weaker H-bond acceptor compared to TfO; (ii) BF_4_ hydration is more sensitive to IL concentration compared to TfO, evidencing modifications on aggregation features; and (iii) strictly connected to (ii) is the key concept of interfacial water—between IL and water domains—playing a role in differentiating the molecular organization of the two IL/water mixtures and their concentration dependence. These features should be kept in mind when analyzing the patterns of density inhomogeneities from SANS experiments.

### Mesoscopic Viewpoint: SANS Experiments

**Figure 4:**
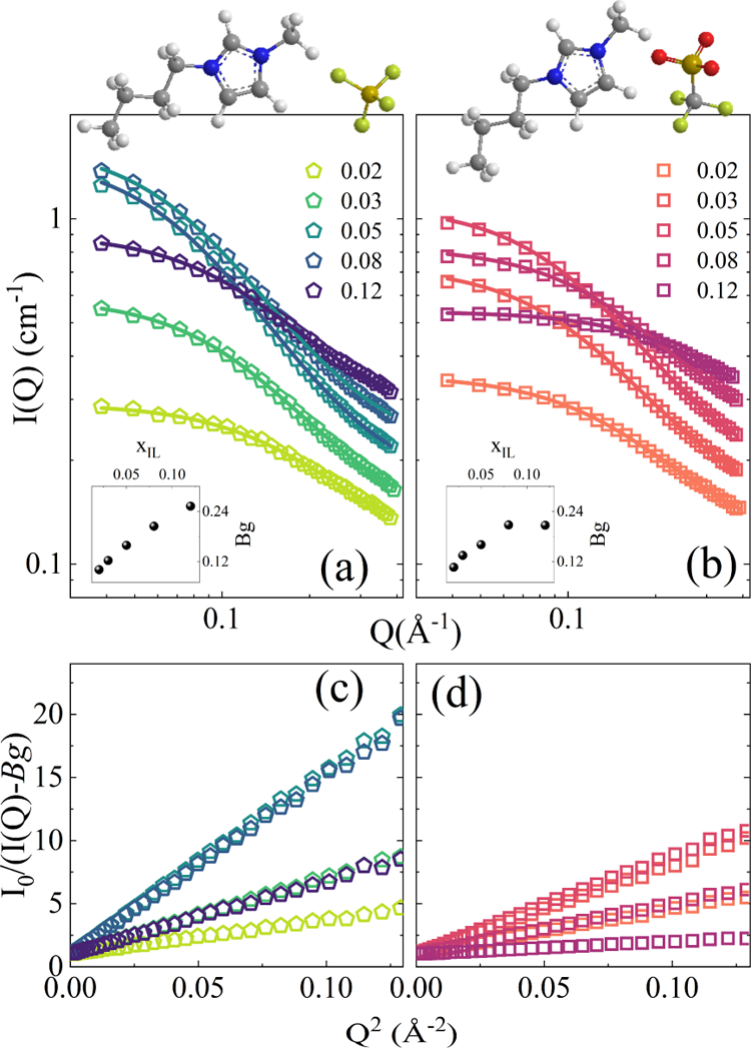
SANS patterns collected on [BMIM][BF_4_] (a) and [BMIM][TfO] (b) aqueous solutions at various molar fraction values and at a temperature of 25 °C. The continuous lines are fitting of the experimental data obtained using eq 1 (see the text for details). Insets: molar fraction dependence of the *B*_g_ contribution for SANS patterns collected on [BMIM][BF_4_] and [BMIM][TfO] aqueous solutions. Panels (c) and (d) show the same experimental data as panels (a) and (b) represented in Ornstein–Zernike-type plots.

SANS profiles obtained for [BMIM][BF_4_]/D_2_O and [BMIM][TfO]/D_2_O solutions in the 0.02 < *x*_IL_ < 0.12 range at 25 °C are shown, as an example, in Figure 4.

The scattering curves were analyzed using the Ornstein–Zernike form for statistical concentration fluctuations given by the equation^16^
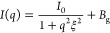
1where *I*_0_ represents the coherent forward scattering intensity, ξ is the short-range correlation length, which measures the decay of density–density correlations, and *B*_g_ represents a constant background. This latter term takes into account the contribution of the incoherent scattering from hydrogen and deuterium atoms, and it can be expressed as
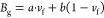
2where *a* and *b* are two constants, while *v*_f_ represents the IL volume fraction, considering the number of hydrogen and deuterium atoms. The *B*_g_ contribution does not depend on temperature, *v*_f_ being constant at a fixed mole fraction. The measurements were collected as a function of temperature, and a global curve-fitting was performed with *B*_g_ as a common parameter. This allowed us to reduce the total number of free parameters in eq 1 during the fitting procedure. The dependence of the parameter *B*_g_ on the IL mole fraction *x*_IL_ is shown in the insets of Figure 4a,b for the SANS patterns collected on [BMIM][BF_4_] and [BMIM][TfO] aqueous solutions. Figure 4 clearly points out that the experimental SANS curves are well reproduced by the Ornstein–Zernike function for all of the examined concentrations. The good agreement between the experimental data and the Ornstein–Zernike behavior, for all of the spectra over the entire studied concentration range, can be inferred also by the Ornstein–Zernike-type plots shown in Figure 4c,d. This suggests that the overall molecular distribution is that expected when strong concentration fluctuations occur.

**Figure 5:**
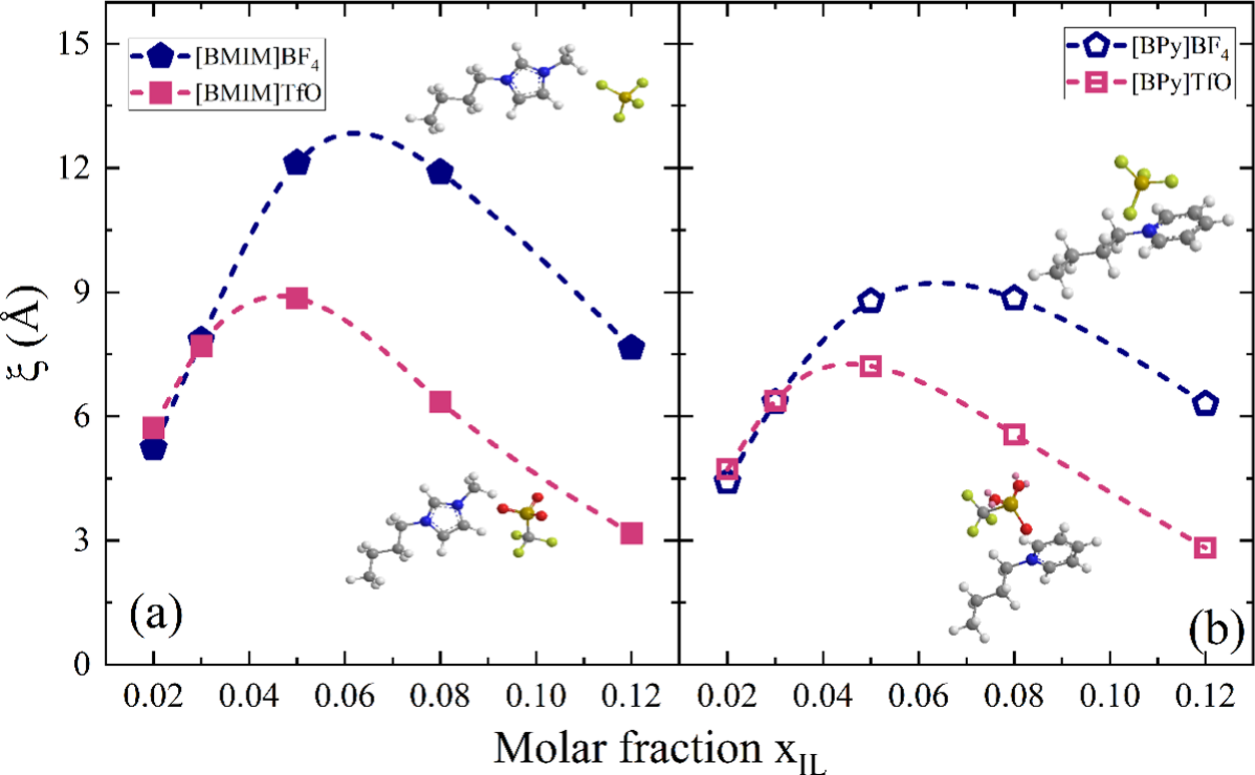
Concentration dependence of correlation lengths ξ estimated for [BMIM][BF_4_] and [BMIM][TfO] aqueous solutions (a) and for [BPy][BF_4_] and [BPy][TfO] aqueous solutions (b) at 25 °C. The dashed lines are guides for the eyes. The error bars, less than 1%, have not been reported for the sake of clarity.

The Ornstein–Zernike correlation lengths estimated as a function of *x*_IL_ for the two imidazolium-based systems and for two pyridinium homologues, [BPy][BF_4_] and [BPy][TfO], are shown in Figure 5a,b, respectively. In this respect, it is interesting to consider how density fluctuations may be sensitive to the replacement of both anion and cation.

In all of the cases, the curves show a maximum at a characteristic IL mole fraction, confirming the density inhomogeneity in water environments. The correlation length ξ for [BMIM][BF_4_]/water mixtures is ∼13 Å, the absolute maximum found in this study. In particular, the trend observed for [BMIM][BF_4_]/D_2_O mixtures is consistent with that reported by Almásy et al.^16^ in previous SANS experiments at *T* = 25 °C, validating the whole data set. The correlation length in the case of [BMIM][TfO] is smaller, ∼9 Å, indicating a lower degree of microheterogeneity. Interestingly, an analogous ξ pattern is observed by comparing [BPy][BF_4_] and [BPy][TfO] mixtures, thus confirming the pivotal role of the anion in driving the solvation/aggregation pathways at these dilution regimes.

**Figure 6:**
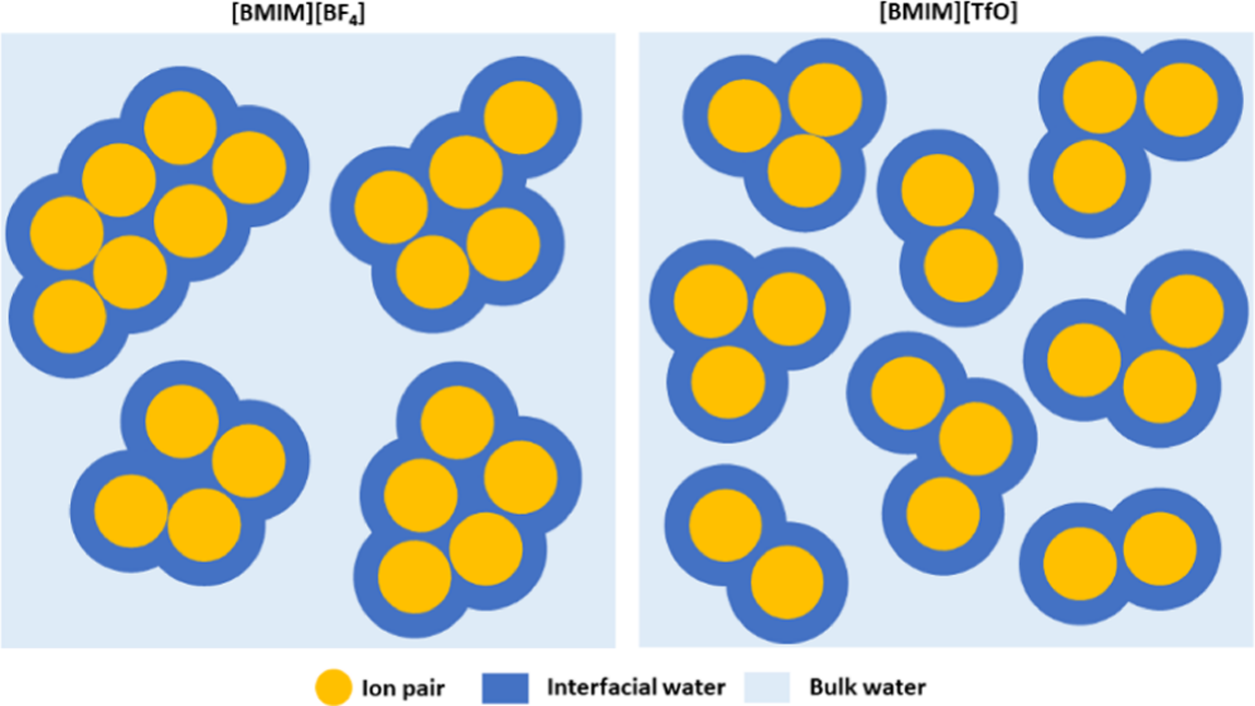
Schematic representation of the molecular distribution (involving IL aggregates, bulk, and interfacial water) expected in a [BMIM][BF_4_]/water mixture (left) and [BMIM][TfO]/water mixture (right), when compared at the same mole fraction, in the water-rich domain. According to experimental SANS and Raman data, the [BMIM][BF_4_] mixture is relatively more heterogeneous with larger ion aggregates and smaller amount of interfacial water.

The SANS results can be related to those obtained from the analysis of SC-UV Raman spectra. Based on these latter, the molecular structure of interfacial water in [BMIM][BF_4_] samples shows a larger concentration dependence than that in [BMIM][TfO] samples, reflecting enhanced modulations of aggregation features. At the same time, SANS experiments suggest that [BMIM][BF_4_] mixtures are also more heterogeneous at mesoscopic length scales and relatively more affected by concentration changes. Presumably, the more stable TfO–water contacts, involving relatively stronger H-bonding interactions than BF_4_–water ones, are relevant in determining the different behavior. In this respect, while hydrophobic interactions triggered by the cations are likely responsible for the occurrence of strong concentration fluctuations and for the general trends depicted in Figure 5, the enhanced IL aggregation, evidenced by Raman data for the [BMIM][BF_4_] mixture (Figure 3), might increase the overall extent of microheterogeneity. As a matter of fact, for both mixtures, the correlation length is similar at low IL concentration, while it increases faster with *x*_IL_ for the [BMIM][BF_4_] mixture, which also shows signatures of ion aggregation (Figures 2 and 3). From a different perspective, it is also possible to infer that the interfacial water itself might regulate (directly or indirectly) the extent of microheterogeneity of the mixtures. The amount of bulk water is different in the two systems just because of the different aggregation propensity of the two anions (Figure 3). A cartoon comparing the molecular distribution in the two mixtures—involving IL aggregates, bulk, and interfacial water—as derived by the analysis of UV Raman and SANS experiments, is sketched in Figure 6. Here, it is emphasized that the [BMIM][BF_4_] mixture is relatively more heterogeneous, encompassing larger IL aggregates and lower amounts of interfacial water.

**Figure 7:**
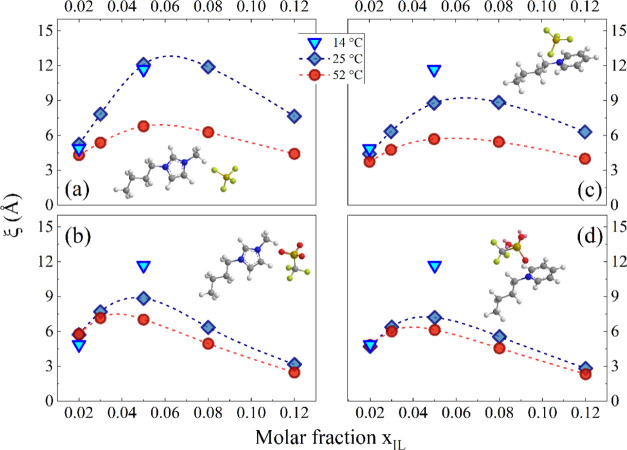
Concentration dependence of correlation lengths ξ estimated for [BMIM][BF_4_] (a), [BMIM][TfO] (b), [BPy][BF_4_] (c), and [BPy][TfO] (d) aqueous solutions at different temperatures. The dashed lines are guides for the eyes.

The temperature dependence of the Ornstein–Zernike correlation length estimated for the four selected ILs as a function of *x*_IL_ is shown in Figure 7.

The plots of Figure 7a show a marked decrease of ξ at 50 °C compared to that at 25 °C in the case of [BMIM][BF_4_] solutions. A similar trend is observed in Figure 7c for the IL sharing the same anion [BPy][BF_4_], although to a lesser extent. Conversely, for the TfO solutions, the plots of Figure 7b,d show small variations of the correlation length on passing from 25 to 50 °C. The increase of the correlation length by lowering the temperature is consistent with the occurrence of critical scattering in the vicinity of the phase separation border, which is reported at ∼8 °C for water-rich [BMIM][BF_4_]/D_2_O mixtures.^53^ The temperature patterns support the interpretation here proposed: the stronger water–anion H-bonds observed in the case of TfO-containing ILs lead to a persistent hydration shell that likely prevents changes of the IL aggregation features (or their mutual interactions) upon temperature changes.

## Conclusions

In this work, a direct spectral subtraction procedure on the OH stretching band of UV Raman spectra made it possible to extract the solute-correlated (SC) spectrum, which highlights spectral contributions due to hydration water in the aqueous solutions of ILs.^34,35^ These SC-UV Raman spectra were determined for aqueous solutions of two related ionic liquids, [BMIM][BF_4_] and [BMIM][TfO], to characterize, for the first time, the local structuring of the interfacial water in microheterogeneous samples, encompassing IL aggregates and bulk water domains. SC-UV Raman data uncovered the different hydration features of [BMIM][BF_4_] and [BMIM][TfO] specifically attributed to anion–water interactions. The existence of IL aggregates and their concentration dependence clearly emerge for [BMIM][BF_4_]. The data are generally consistent with the idea that in the water-rich regime ILs, similarly to surfactants, tend to associate through their hydrophobic portions. Yet, specific features arise due to the nature of the anion. In this respect, SC-UV Raman spectra evidenced how ionic aggregates enlarge at increasing IL concentrations in the case of [BMIM][BF_4_], which forms relatively weak H-bonds with water. On the other hand, stronger water–anion H-bonds are observed for [BMIM][TfO], leading to a more persistent hydration shell that likely prevents changes of the IL aggregates (or their mutual interactions). Overall, SC-UV Raman spectroscopy allowed us to obtain the spectral distribution of hydration water in prototypical ILs. This is a step forward to achieving a deep view of the H-bonding interactions that establish between interfacial water and ionic liquids in the water-rich regime.

SANS experiments, also extended to [BPy][BF_4_] and [BPy][TfO] aqueous solutions, evidenced the occurrence of significant concentration fluctuations and microinhomogeneity for all of the IL/water mixtures in the water-rich domain. These were mainly ascribed to the IL aggregation, likely induced by the hydrophobic parts of the cation. Nevertheless, the major variations of concentration fluctuations observed for [BMIM][BF_4_] (and [BPy][BF_4_]) compared to those of the TfO analogues led to the idea that the weaker anion–water interactions, favoring IL aggregation, contribute to enhancing the microheterogeneous character of the mixture.

In conclusion, UV Raman and SANS experiments allowed us to probe at molecular and mesoscopic scales, respectively, how concentration-induced changes in the local structure of the anion hydration shell (interfacial water), in a water-rich environment, relate to modifications of the microheterogeneity (concentration fluctuations) in IL/water mixtures.
